# Analysis of Context Dependence in Social Interaction Networks of a Massively Multiplayer Online Role-Playing Game

**DOI:** 10.1371/journal.pone.0033918

**Published:** 2012-04-04

**Authors:** Seokshin Son, Ah Reum Kang, Hyun-chul Kim, Taekyoung Kwon, Juyong Park, Huy Kang Kim

**Affiliations:** 1 Multimedia and Mobile Communications Laboratory, School of Computer Science and Engineering, Seoul National University, Seoul, Republic of Korea; 2 Graduate School of Information Security, Korea University, Seoul, Republic of Korea; 3 Department of Computer Software Engineering, Sangmyung University, Cheonan, Republic of Korea; 4 Department of Physics, Kyung Hee University, Seoul, Republic of Korea; INSERM & Universite Pierre et Marie Curie, France

## Abstract

Rapid advances in modern computing and information technology have enabled millions of people to interact online via various social network and gaming services. The widespread adoption of such online services have made possible analysis of large-scale archival data containing detailed human interactions, presenting a very promising opportunity to understand the rich and complex human behavior. In collaboration with a leading global provider of Massively Multiplayer Online Role-Playing Games (MMORPGs), here we present a network science-based analysis of the interplay between distinct types of user interaction networks in the virtual world. We find that their properties depend critically on the nature of the context-interdependence of the interactions, highlighting the complex and multilayered nature of human interactions, a robust understanding of which we believe may prove instrumental in the designing of more realistic future virtual arenas as well as provide novel insights to the science of collective human behavior.

## Introduction

It has been recently reported that around 40% of Internet users play some form of an online game (http://www.develop-online.net/news/36618/40-of-all-internet-users-play-online-games). Among the games, Massively Multiplayer Online Role-Playing Games (MMORPGs for short), perhaps the most sophisticated and complex, are known to be enjoyed by a dedicated base composed of no less than 20 million people worldwid (http://www.brighthub.com/video-games/mmo/articles/35992.aspx). An MMOPRG typically features a real world-like arena set in a fantastical age in which gamers engage in a variety of interactions with other players through battles and commerce (e.g., exchange or sales of valuable items), or purely recreational activities. The existence of a large dedicated fan base is attributed to the engrossing and persistent nature of MMORPGs, the players being able to groom their characters over a time span of many months or years.

Given that the complexity and longevity of the user experiences in MMORPGs now rival the real life, it is natural to anticipate the complete digital record of players’ activities in MMORPGs to present a highly promising opportunity to study and understand in depth the patterns and dynamics of complex human behavior. Such prospects are not restricted to MMORPGs; many other large-scale data sets representing human activities and dynamics such as mobile communication records are the focus of active scientific research. Although many service providers, mostly private firms, may still be reluctant to share data they gathered for research out of concerns for privacy and security reasons, collective efforts at analyzing massive data by the industry and the academia are being increasingly called upon and being vigorously pursued in the hopes of uncovering new insights that would potentially benefit both parties [1]–[4].

**Figure 1:**
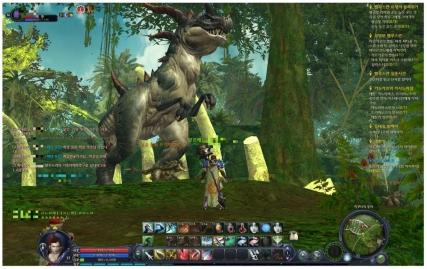
An in-game screen shot from AION, a popular MMORPG. AION’s design puts heavy emphasis on cooperation for success in gaming, generating rich and detailed data of collaborative human interactions. Copyright NCSoft, Inc.

Our research presented here constitutes another example: In collaboration with NCSoft, Inc., a leading global online game services provider, we analyzed a comprehensive data set containing nearly all in-game user activities from AION, one of their staple MMORPGs. Any information that might reveal the users’ true identities (real names, messages, or locations via IPs of their terminals) were not made available to us. Upon its launch in November of 2008 AION was praised for its quality, and as of early 2011 it ranks as the second-most played MMORPG with over three million subscribers in more than sixty countries (http://www.etnews.co.kr/news/detail.html?id=201011100087). In a fantastical yet realistic setting (Figure 1), players of AION engage in social interactions or develop their in-game characters by completing quests or winning battles. While solitary play is certainly possible, activities involving multiple users, such as social (e.g. communication) or collaborative (e.g. mission-oriented community formation) are the most prevalent, and any newcomer soon finds out that cooperation with other players is indispensable for any meaningful achievements. This fact that social interactions with others is essential for a gamer’s success has prompted us to utilize the framework of network science that has garnered much attention recently as a useful technique for modeling and analyzing complex interacting systems [5]–[8], as the human interaction data can be naturally represented as a network with people as the nodes and interactions between them as the edges. Specifically, we focused on the nature of the context-dependent interplay between various types of interactions between AION gamers: Using several network concepts and measures, we compared six distinct networks representing six most popular types of interactions from the AION log. From this we demonstrate how the microscopic nature and context of various interactions lead to large-scale network properties of the interaction networks and dictate their correlations in a significant way, the understanding of which we believe is essential, as our real-world experience are also composed of many distinct interactions with various levels of correlation. [4], [9].

## Materials and Methods

### Ethics Statement

This study was granted a waiver of ethics review by the Institutional Review Board of Kyung Hee University on the following grounds: the anonymity of users in the data we were provides; and that the users had agreed, via an on-line End User License Agreement upon joining AION, to grant NCSoft, Inc. full permission to use and share their data for analysis with parties of NCSoft’s choosing, one of which is group of authors.

Our AION data list all in-game actions taken by its users for a total of nearly three months (eighty seven days, between April 10th and July 5th of 2010), composed of over 1.5 million entries that list user-to-user interactions between a total of 68,309 users. Each entry lists a Sender (S) and Receiver (R) player pair and the interaction type, of which the following six are the most prevalent and were thus considered in our analysis:

1. Friendship (abbreviated F, constituting 6.6% of interactions): S adds R to his Friend List.
2. Private Messaging (PM, 11.3%): S sends R a private message. PM can take place between any two players (they do not need to be on each other’s Friend Lists) that are online only. S, while online, must use Mail (see below) to send a message to a player who is offline.
3. Party Invitation (PI, 58.4%): S invites R to join his **Party**, a group of up to six players often created for collaborative play of a short duration. A Party is not permanent, and can be easily disbanded. Membership in a party grants special abilities for cooperation such as an exclusive members-only communication channel.
4. Trade (T, 11.5%): S requests R an exchange of items, which R then agrees to. S can initiate a Trade with anybody by approaching them in-game and asking if they are interested. Compare with Shop below.
5. Mail (M, 11.0%): S sends a mail to R. Unlike private messaging, R does not need to be online to receive mail.
6. Shop (S, 1.2%): S buys an item from R who is in the **Merchant mode**. A player in the Merchant mode is someone who has set up a temporary shop for the sole intention of selling one’s items; they can publicly announce their inventory and prices, and can communicate with others (often for inventory checking or price negotiation, if they choose to). While in the Merchant mode, however, the player is immobile, and thus cannot engage in any action that requires movement such as hunting, battles, etc.

**Figure 2:**
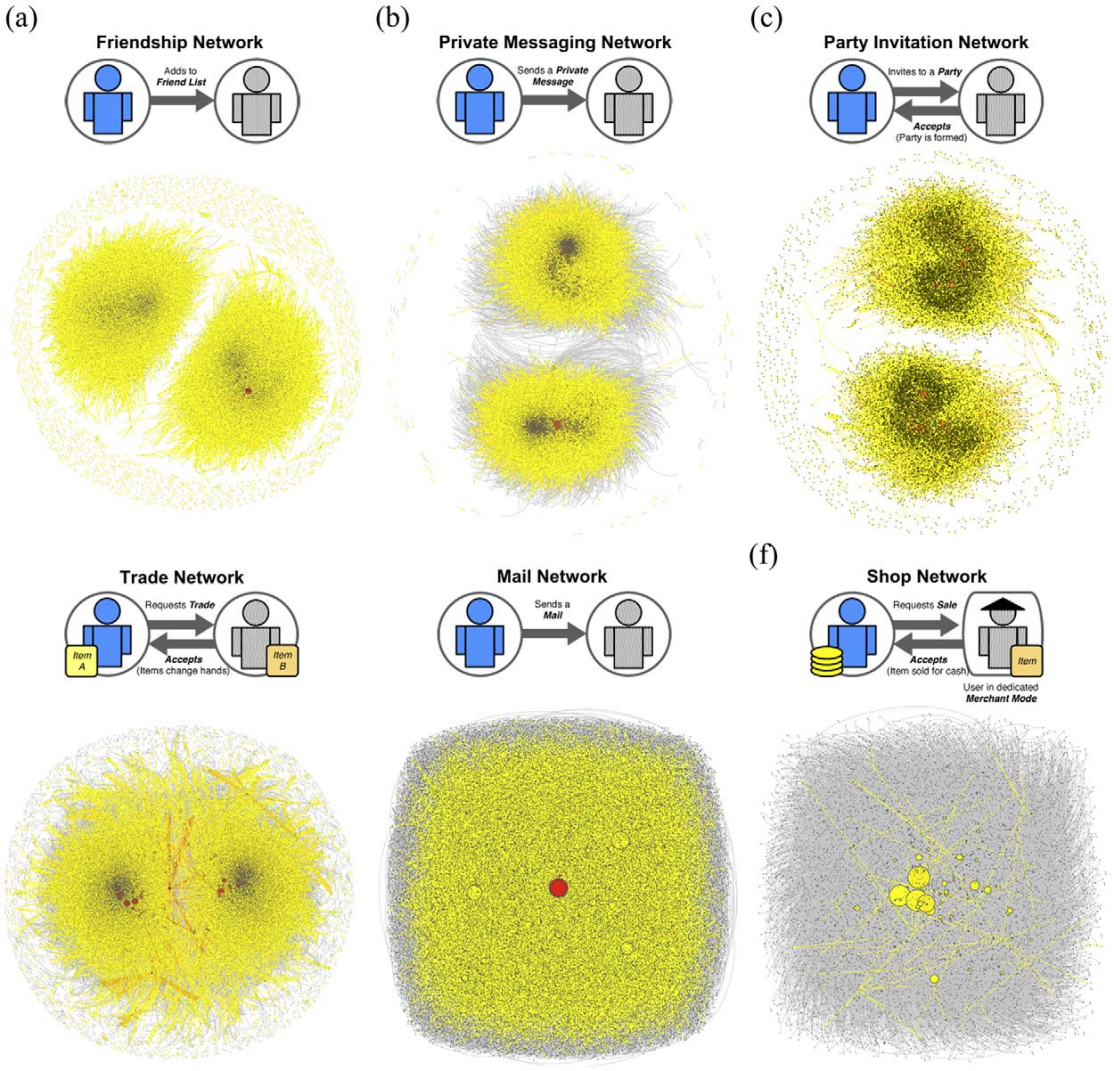
The definition and the graphic representation of the six AION networks. The Realm-vs-Realm design of AION where gamers belong to one of two tribes (Heavenly and Diabolical) that cannot communicate is evident in the existence of two similarly-sized large components. Red-colored nodes indicate exceptionally high-degree nodes.

Since all interactions involve two people, they define distinct social networks composed of directed edges from S to R (shown in Figure 2), conferring on us a unique opportunity to compare the implications of the nature of interactions on the global network structure. A recent noteworthy study in a similar vein was presented by Szell *et al.* where they studied the relationship between structural balance and friendly/hostile interactions [4], [10]. Based on their work, our study goes further by considering a larger network data set containing more interaction types. This allows for an investigation into the general aspects of correlations observed between different interactions that are not only exclusive (e.g. friendly versus hostile) but that may be combined by the player to constitute a specific course of action, as we discuss later in the case of communication and trading/shopping of items.

To characterize the structures of and correlations between the interaction network, we measured the following quantities [8]:

- The **node degree distribution**, one of the most basic network measures, is known to correlate with many (but not all) properties of the network. The degree (often denoted 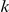) of a node is the number of nodes connected to it, called its neighbors. In directed networks as ours there are two degree types, the in-degree 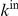 (the number of edges pointing at the node) and the out-degree 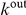 (the number of edges pointing from the node). Also in a directed network, a connected node pair 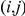 is called **reciprocal** if there exist edges pointing in both directions. The **reciprocity** of a network is the fraction of reciprocal node pairs among all connected node pairs.
- Two nodes are said to belong to the same **component** if there exists a path, a series of connected nodes, between the two. Networks typically exhibit a single predominantly large component called the Giant Connected Component (GCC). The length of the shortest path between two nodes is called the shortest distance between the two. The **diameter** of a network is the largest of the shortest paths.
- The **clustering coefficient** 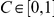 is defined as the probability that two neighbors of a node are themselves neighbors, and thus represents the relative abundance of triangles in networks. More generally in a directed network a triplet of nodes can possess a richer structural details, and the **triad census** of the thirteen distinct configurations or motifs are often carried out [11], [12]. The benchmark for the relative abundance or scarcity of a motif is, naturally, the null model (random graph). Specifically, the relative frequency of each the thirteen motifs against their expected number in the null model is quantified via the Z-score.

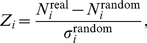
(1)where 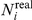 is the number of motif 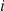 found observed in the network, and 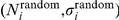 are the expected number and its standard deviation in the randomized network [11]. Often, as in this paper, the normalized version 
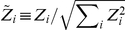
 is used.
- Finally, we study the similarities between networks in order to find how they are correlated. We believe this analysis to exhibit the true relationships between the nature of the various interactions, not always evident from the examination of global summary statistics discussed above. For instance, two networks can show similar values of clustering, yet that does not at all guarantee that nodes connected in one network are connected in the other, or that the nodes show similar levels of activity. Thus we here consider two measures of network overlap. The first is the **Link Overlap** between two networks 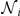 and 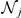 quantified by the Jaccard coefficient.

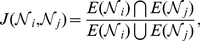
(2)where 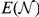 notes the edge set of network 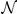. The second is the **Degree Overlap**, given by the Pearson Correlation Coefficient (PCC) between the node degrees in pairs of networks.

**Table 1:** Basic network characteristics of AION interaction networks and other popular social networks.

Networks	Number of Nodes	Number of Links	Average Degree	Diameter/Average Path Length in GCC	Clustering Coefficient /Ratio to Random Network
Friendship	29,995	103,437	13.5	15/4.80	0.035/81.6
Private Messaging	20,107	176,245	26.2	11/3.81	0.035/25.7
Party Invitation	45,590	910,171	43.8	15/3.90	0.070/72.9
Trade	45,567	179,277	9.6	27/5.70	0.051/266.5
Mail	56,040	170,774	6.8	13/7.63	0.001/8.2
Shop	9,423	18,882	4.0	13/6.85	0.004/10.2
Facebook	63,730	817,090	25.7	NA	0.22/NA
Wikipedia	1,870,709	36,532,531	39.1	NA	0.23/NA
Flickr	2,302,924	22,838,276	20.9	NA	0.18/NA
YouTube	3,223,588	9,386,594	5.8	NA	0.09/NA
Cyworld	11,537,961	177,566,730	30.9	NA	0.16/NA

Basic network properties of the six interaction networks from AION, compared with some other well-known networks (Facebook, Wikipedia, Flickr, YouTube, and Cyworld, data taken from [16]). All AION interaction networks show common properties such as the small network diameter and average path lengths in their giant connect component.

## Results

### A. Basic Network Characteristics

**Figure 3:**
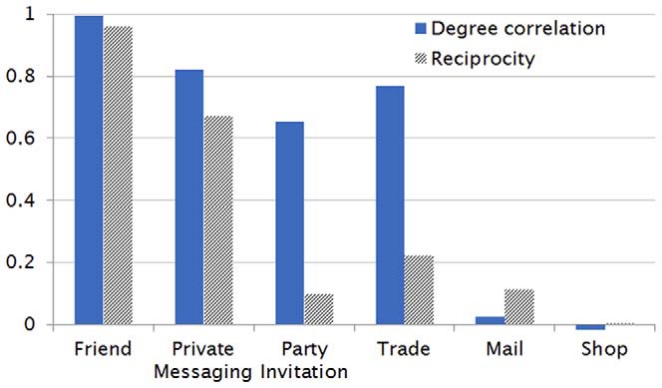
The Pearson correlation coefficient between the in- and out-degrees of nodes (solid), and the reciprocity of edges in the AION interaction networks. The four social-type networks (F, PM, PI, and T) can be further divided according to the reciprocity, the low value of which in Party Invitation and Trade interactions are believed to indicate significant strategizing in the latter two cases.

In Table 1 we present the basic undirected characteristics of each network from AION. It also contains characteristics of other well-known networks for comparison purposes. First, we see that the diameters of networks are small, indicative of the “small-world” property (see Table S1). While the clustering coefficients for the network in AION are smaller than what are usually found in typical social networks [13], four networks – Friendship, Private Messaging, Party Invitation, and Trade – do show much higher relative abundance of triangles than random networks with same size (nodes and edges) by a a factor of 25 or larger, while for Mail and Shop it is noticeably less so. Thus in the following we shall call the four (F, PM, PI, and T) networks “social-type” networks for convenience. When we examine the PCC between the in- and out-degrees on nodes (Figure 3), we see that the four relatively highly clustered networks (F, PM, PI, T) show high values, indicating that the ones who initiate these interactions actively are also like to be invited to them. When we inspect the reciprocity of the interactions, however, we find interesting differences among the four networks: as we also see in Figure 3, whereas Friendship and Private Messaging are highly reciprocated, Party Invitation and Trade Initiations are generally not.

We believe that this demonstrates an interesting differences in the nature of the activities, even though they belong to the broad class of “social interactions” in common parlance. First, it is expected that casual or truly interactive actions such as Friendship and Private Messaging (i.e. conversations) are highly reciprocal, both by common sense and literature. The low reciprocity of the latter two networks (PI and T), therefore, reflect their fundamental differences. We believe that one possible explanation is the level of strategizing involved in making such interactions: since a gamer inviting others to form a Party means that one is expecting the invitee to be helpful in concrete terms (items or money), it is possible that one seeks stronger or more experienced players than oneself, leading to the observed low reciprocity. A similar explanation may be applied to the case of the Trade network, where the strength of a player is plausibly reflected in the items that one carries. While a full-fledged treatment is out of the scope of this work, there also exists a sizable volume of literature on the complex nature of “exchange networks” as significant underlying foundation of social structure [17]–[19].

**Figure 4:**
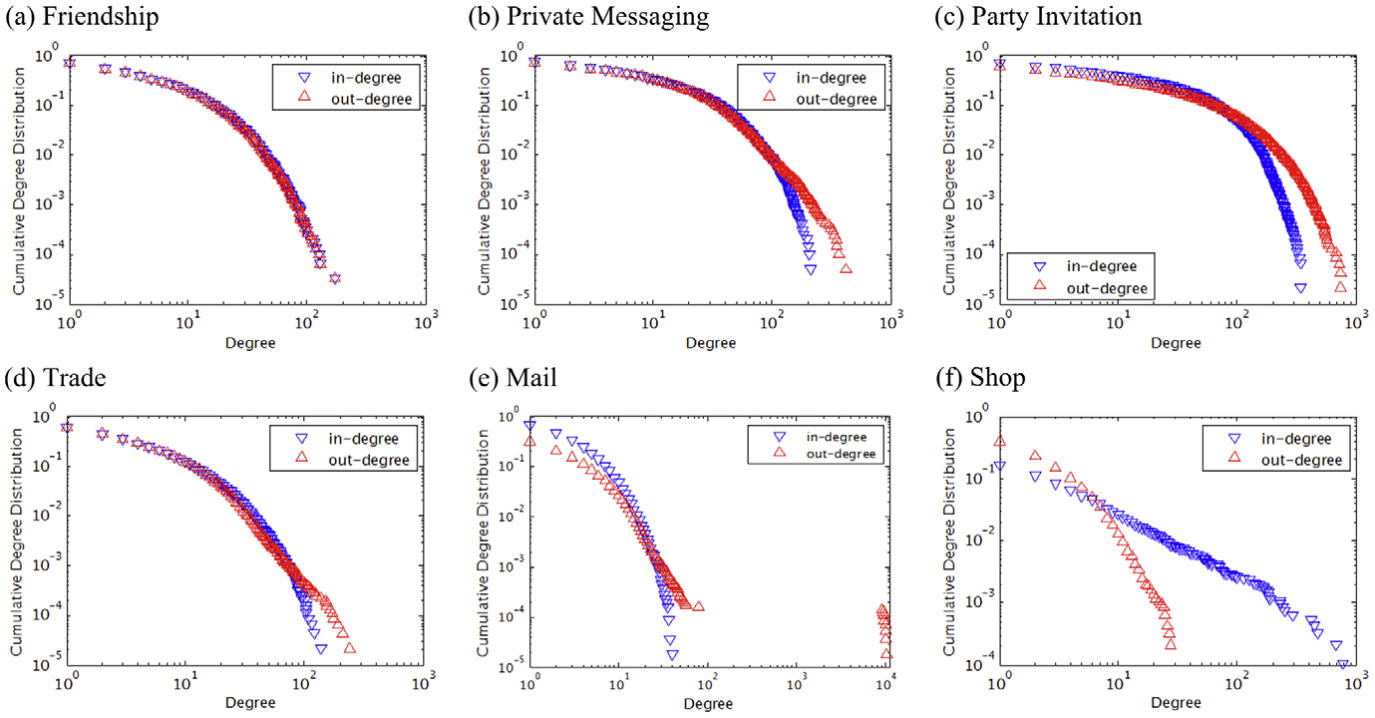
The in- and out-degree distributions in AION are most dissimilar in the case of the Mail and Shop networks. In Shop, the highly skewed in-degree distribution is caused by the existence of œmagnate merchants In Mail, the outliers in the out-degree nodes specify a special class of high-volume senders such as in-game managers.

The nature of interactions affecting the network properties can also be observed in the case of the Shop and the Mail networks. The most noteworthy here is the low correlation between the in- and the out-degrees. Since in the Shop interaction the out-degree means the buyer and the in-degree the merchant, the small correlation implies that there exists a strong tendency for role specialization among gamers into *magnate merchants* and others. A similar effect is present in the Mail network also: Mail is the only method of gamer-to-gamer communication that works offline, and from this we assume that a small number of gamers develop into high-volume mail senders (since we do not have access to the messages, at this point we were unable to discern the exact nature of high-volume Mailers.).

Our discussions thus far render the degree distributions, presented in Figure 4, straightforward to understand: First, the similarities between the in- and the out-degree distributions in the F, PM, PI, an T networks are consistent with the high level of correlation between the two. In Shop and Mail, the two are clearly disparate: in Shop, the “magnate traders” appear as the high in-degree nodes, while in Mail the high-volume mail senders are the high out-degree nodes.

### B. The Triad Census

**Figure 5:**
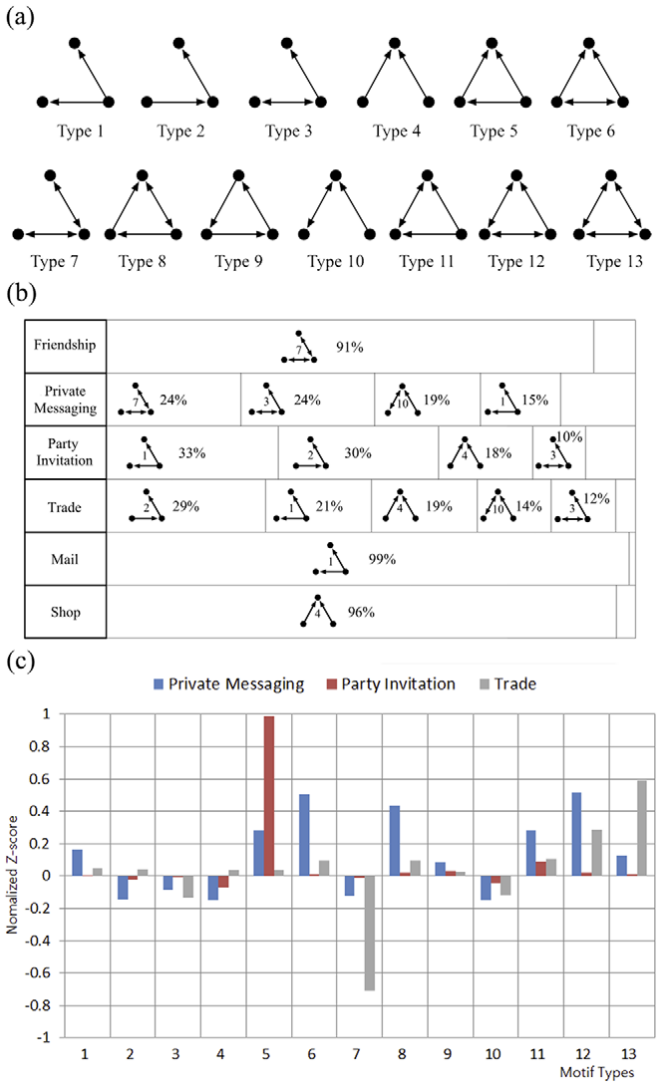
Network motif analysis of node triplets reveal detailed interactions patterns in directed networks. (a) The thirteen possible motifs composed of three nodes in a directed network. (b) The fractions of each motif type in each of the six networks. Motifs accounting for fewer than 10% of the motifs are not shown. Friendship, Mail, and Shop each show one dominant motif, consistent with the high or low reciprocity found in the networks. (c) A closer look at the (normalized) Z-score triad census of Private Messaging, Party Invitation, and Trade networks where no dominant motif is evident, we used the Z-score method is employed to determine significantly over- and underrepresented triangular motifs. Overrepresented motifs (with 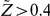) are indeed closed triangles, consistent with the relatively high clustering tendencies in these networks.

As discussed earlier, the relative prevalence of each of the thirteen triad network motifs given in Figure 5 (a) tell us in more detail the interaction patterns in networks. For our AION networks, we show them in Figure 5 (b), in terms of both their fraction and the Z-scores assessed against the null model (Eq. (1), also see Tables S2 and S3). Interestingly, the Friendship, Mail, and Shop networks each show one predominant motif type: for instance, in Friendship network type 7 account for more than 90% of node triplet relationships, which can be attributed to the highly reciprocal nature of the interactions. The opposite reasoning can be applied to Mail and Shop: low reciprocity reflects again the existence of high-volume senders and magnate traders. Comparing the prevalence of motifs against the null models allows us to detect signals discounted by random expectations, and this is done via Z-scores (Eq. (1)). This is particularly necessary and illuminating in the cases of the other three networks (Party, Private Messaging, and Trade), since by considering the null models we can see that even though multiple motifs can be similarity abundant (Figure 5 (b)), some can be over- or under-represented in a significant manner, as we see in Figure 5 (c). Finally, we note that the overrepresented ones (i.e., ones showing positive Z-scores) are the closed triangle ones in all these network, reflecting the relatively high clustering tendencies in the social-type networks. Yet, among the triangular motifs types 6 and 8 are conspicuously absent in the Party Initiation network, consistent with the low level of reciprocity in the networks.

### C. Network Overlap

**Figure 6:**
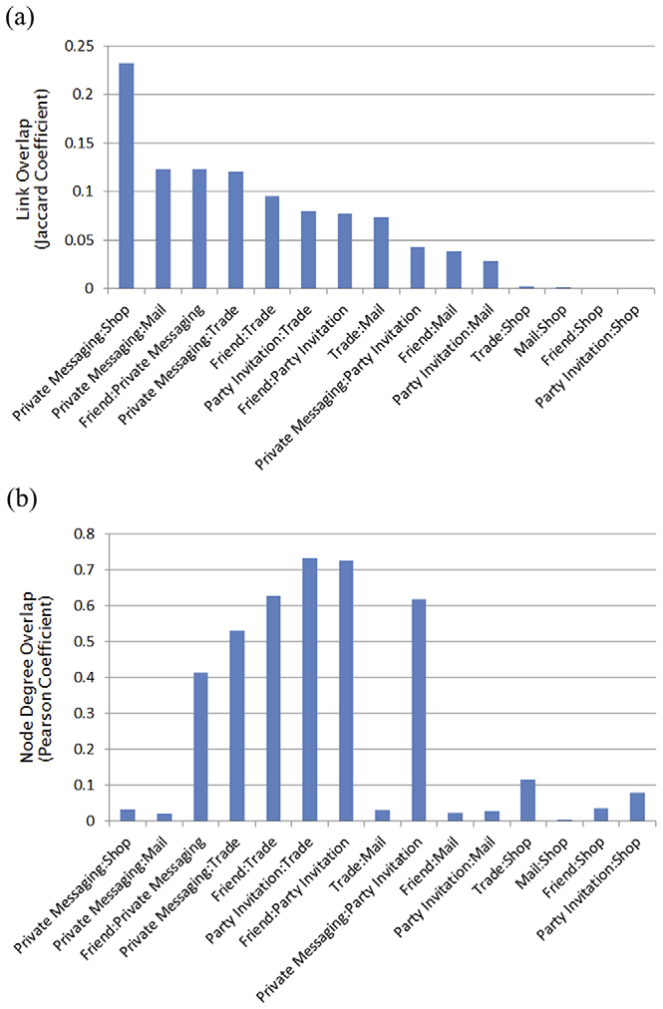
Pairwise network overlaps indicating the similarity or dependence between interactions. (a) The link overlap. The largest link overlap is found between the Private Messaging and the Shop networks, reflecting the fact that private messaging (for bargaining) nearly always precedes the sales of items via the Shop interaction. (b) The node overlap quantifying the node degree overlap between different networks. High degree overlaps occur between the four social-type networks, indicating that many gamers make a fair mix of the actions. The low degree overlaps in different pairs indicate the role specialization discussed in the text.

The results for the network overlaps (Link and Degree Overlaps) for all fifteen possible network pairs are given in Figure 6. Examining the link overlap (Figure 6 (a)), we find the Shop network most interesting: while it shows the highest link overlap Private Messaging (in fact, the highest among any network pair), that with any other network is negligibly small. This is a result of the fact that users often engage in conversations when shopping, most often for inventory checking and price bargaining (as we often do in real life), even though it is not mandatory: one can simply pick up an item to buy and pay the asking price, while the low overlap with the other social-type interactions is the result of the existence of magnate merchants so that Shop transactions commonly take place between gamers with no particular social or personal relationships. The node degree overlap (Figure 6 (b)) is another way of seeing the connection between interactions: here, for instance, the Party Initiation and the Trade networks show a positive PCC value exceeding 0.7, which can be understood by the fact that a Party activity, being above all the favorite way of engaging in battles or hunting, often concludes with members Trading booties.

## Discussion

In this paper, we studied and compared large-scale multi-relational user interactivity networks representing various types of interactions in AION. Utilizing the framework of network science, we measured and discussed how the the local and the global properties of the networks correlate with the detailed nature and context of the interactions. While so far it is still commonplace in network studies to treat all links as being of the same type (whether they are truly so, or different but similar enough to do so), our work shows that when available data contain enough detail on the different edge types we can find nontrivial and frequently drastically different patterns emerge in their global characteristics. We also discussed some noteworthy cases in which we could present plausible explanations of the observed differences to the way in which various interaction types have to be enacted in a certain way (e.g., Shop and Private Messaging) or the active strategizing of gamers given the reality of asymmetry in gamer strengths or aptitudes (e.g. Party Invitation and Friendship).

We can envision several possible avenues for interesting and useful research based on our findings in this work. One is a sophisticated framework for profiling users based on the pattern of the combinations of various activities each has taken – e.g., the relative fraction of the activities, the ordering in which they were taken, and the directionality of in the activities. This has potentially very useful practical implications as well as scientific: “game bots,” semi-automatic softwares that can play MMORPGs are serious concerns to service providers because they can ruin the gaming experience for paying customers – the loss of revenue incurred due to such activities is said to be over tens of millions of US dollars (for instance, see http://news.bbc.co.uk/2/hi/technology/7645059.stm for the case of Blizzard Entertainment, Inc., provider of another popular online game World of Warcraft. NCSoft’s own estimates also tally up to many million USD over the past several years – and cause serious bias in data. Node activity profiling is expected to help filter out these unwanted game bots that are often employed to carry out menial, repetitive tasks from humans who are expected to have a more balanced and evenly mixed activity profile [14]. Another possible avenue of interesting research is a full-fledged validation of various network algorithms and models being devised and proposed at a face pace. For instance, we anticipate the full record of explicit memberships in Parties in AION to be very helpful in validating various “community detection” algorithms, contributing to the advancement of the understanding of modular structures in networks [13], [15]. We believe that our work constitutes merely an early step in exploring the rich detail in comprehensive, high-quality data from MMORPGs that are bound to become more accessible, and anticipate interesting and fruitful research to take place that enrich our understanding of complex human dynamics.

## Supporting Information

Table S1 **Network diameters from 100 randomized versions of networks.**
(PDF)

Table S2 **Complete frequency distribution for triangular motifs.**
(PDF)

Table S3 **Complete normalized Z-scores for triangular motifs.**
(PDF)
