# Supplementary material for: Analysis of Context Dependence in Social Interaction Networks of a Massively Multiplayer Online Role-Playing Game
Source: PLoS One. 2012 Apr 4;7(4):e33918. doi: 10.1371/journal.pone.0033918 (PMC3319537; doi:10.1371/journal.pone.0033918)
Supplement: Table S1 — Network diameters from 100 randomized versions of networks. (PDF) [file pone.0033918.s001.pdf]

Supporting Information

S Son, A R Kang, H Kim, T Kwon, J Park, H K Kim

Table S1. Network diameters from 100 randomized versions of networks.

network	mean (stdev) diameter from 100 random samples
Friendship	11.09 (0.60)
Mail	9.99 (0.67)
Party Invitation	7.48 (0.52)
Private Messaging	8.00 (0.37)
Shop	11.53 (0.82)
Trade	10.89 (0.60)
