# Supplementary material for: Analysis of Context Dependence in Social Interaction Networks of a Massively Multiplayer Online Role-Playing Game
Source: PLoS One. 2012 Apr 4;7(4):e33918. doi: 10.1371/journal.pone.0033918 (PMC3319537; doi:10.1371/journal.pone.0033918)
Supplement: Table S2 — Complete frequency distribution for triangular motifs. (PDF) [file pone.0033918.s002.pdf]

Supporting Information

S Son, A R Kang, H Kim, T Kwon, J Park, H K Kim

Table S2. Complete frequency distribution for triangular motifs.

	Party Invitation	*Friendship*	*Private Messaging*	*Trade*	*Mail*	*Shop*
Type 1	32.66%	0.67%	14.90%	20.76%	99.71%	2.01%
Type 2	29.69%	0.17%	8.40%	29.16%	0.05%	1.70%
Type 3	10.17%	3.25%	23.57%	12.33%	0.12%	0.01%
Type 4	17.84%	0.13%	8.41%	19.00%	0.05%	96.13%
Type 5	1.44%	0.00%	0.18%	0.31%	0.02%	0.13%
Type 6	0.27%	0.00%	0.14%	0.17%	0.00%	0.00%
Type 7	0.87%	91.21%	24.08%	3.35%	0.01%	0.00%
Type 8	0.22%	0.00%	0.11%	0.26%	0.00%	0.00%
Type 9	0.16%	0.00%	0.01%	0.06%	0.00%	0.00%
Type 10	6.32%	3.37%	19.48%	13.64%	0.03%	0.01%
Type 11	0.17%	0.01%	0.12%	0.20%	0.01%	0.00%
Type 12	0.15%	0.06%	0.34%	0.49%	0.00%	0.00%
Type 13	0.04%	1.12%	0.28%	0.26%	0.00%	0.00%
