# Supplementary material for: Analysis of Context Dependence in Social Interaction Networks of a Massively Multiplayer Online Role-Playing Game
Source: PLoS One. 2012 Apr 4;7(4):e33918. doi: 10.1371/journal.pone.0033918 (PMC3319537; doi:10.1371/journal.pone.0033918)
Supplement: Table S3 — Complete normalized Z-scores for triangular motifs. (PDF) [file pone.0033918.s003.pdf]

Supporting Information

S Son, A R Kang, H Kim, T Kwon, J Park, H K Kim

Table S3. Complete normalized Z-scores for triangular motifs.

	Party invitation	*Friendship*	*Private messaging*	*Trade*	*Mail*	*Shop*
Type 1	0.00	0.00	0.16	0.05	0.00	-0.01
Type 2	-0.02	-0.04	-0.14	0.04	0.00	-0.01
Type 3	-0.01	0.05	-0.09	-0.14	-0.03	-0.01
Type 4	-0.07	-0.06	-0.15	0.04	0.00	0.01
Type 5	0.99	0.07	0.28	0.04	0.00	0.01
Type 6	0.01	0.19	0.50	0.09	0.02	0.01
Type 7	-0.01	-0.54	-0.12	-0.71	-0.99	-0.98
Type 8	0.02	0.24	0.44	0.10	0.08	0.01
Type 9	0.03	0.00	0.08	0.03	0.00	0.00
Type 10	-0.04	-0.03	-0.15	-0.12	-0.01	-0.22
Type 11	0.09	0.26	0.28	0.11	0.00	0.00
Type 12	0.02	0.42	0.51	0.29	0.07	0.00
Type 13	0.01	0.60	0.12	0.59	0.06	0.00
